# The Association of Thyroid-Stimulating Hormone (TSH) Levels and Lipid Profile in Euthyroid Patients with Familial Hypercholesterolemia

**DOI:** 10.1155/2023/4711275

**Published:** 2023-05-16

**Authors:** Maryam Heidarpour, Parastesh Rezvanian, Mohammad Amin Sadri, Parsa Keshavarzrad, Rezvan Zakeri, Omid Vakilbashi, Davood Shafie, Masood Shekarchizadeh, Sonia Zarfeshani, Najmeh Rabbanipour, Jamshid Najafian, Golnaz Vaseghi, Nizal Sarrafzadegan

**Affiliations:** ^1^Isfahan Endocrine and Metabolism Research Center, Isfahan University of Medical Sciences, Isfahan, Iran; ^2^Applied Physiology Research Center, Cardiovascular Research Institute, Isfahan University of Medical Sciences, Isfahan, Iran; ^3^Isfahan University of Medical Sciences, Isfahan, Iran; ^4^Heart Failure Research Center, Cardiovascular Research Institute, Isfahan University of Medical Sciences, Isfahan, Iran; ^5^Isfahan Cardiovascular Research Center, Cardiovascular Research Institute, Isfahan University of Medical Sciences, Isfahan, Iran; ^6^Hypertension Research Center, Cardiovascular Research Institute, Isfahan University of Medical Sciences, Isfahan, Iran; ^7^School of Population & Public Health, University of British Columbia, Vancouver, BC, Canada

## Abstract

Previous studies reported a relationship between thyroid-stimulating hormone (TSH) and low-density lipoprotein cholesterol (LDL-C) levels. In this study, we aim to evaluate the impact of TSH levels on lipid profile in patients with familial hypercholesterolemia (FH) and euthyroid state. Patients were selected from the Isfahan FH registry. The Dutch Lipid Clinic Network (DLCN) criteria are used to detect FH. Patients were classified into no FH, possible FH, probable FH, and definite FH groups based on the DLCN scores. Patients with any cause of secondary hyperlipidemia, including hypothyroidism, were excluded from this study. The study group consisted of 103 patients with possible FH, 25 patients with definite FH, and 63 individuals with no FH. The mean TSH and LDL-C levels among participants were 2.10 ± 1.22 mU/l and 142.17 ± 62.56 mg/dl, respectively. No positive or negative correlation was found between serum TSH and total cholesterol (*P* value = 0.438), high-density lipoprotein cholesterol (*P* = 0.225), triglycerides (*P* value = 0.863), and LDL-C (*P* value = 0.203). We found no correlation between serum TSH levels and lipid profiles in euthyroid patients with FH.

## 1. Introduction

Familial hypercholesterolemia (FH) is a common inherited disorder attributable to mutations in low-density lipoprotein receptor (*LDLR*), apolipoprotein B (*ApoB*), proprotein convertase subtilisin/kexin type 9 (*PCSK9*), and low-density lipoprotein receptor adaptor protein 1 (*LDLRAP1*), which results in high serum level of low-density lipoprotein cholesterol (LDL-C) [1]. FH is subdivided into heterozygous (with a prevalence of nearly 1 : 250) and homozygous (with a prevalence of 1 : 250000) forms. Physical signs and symptoms of this disease include tendon xanthomas, arcus cornea, and xanthelasma. The diagnosis is based on laboratory manifestations and if-needed genetic analysis in patients identified by cascade screening [2]. FH patients are at a high risk of cardiovascular diseases (CVD) like myocardial infarction (MI) due to the deposition of cholesterols leading to atherosclerotic plaques [3].

Besides the diverse causes of high LDL-C concentration, the serum thyroid-stimulating hormone (TSH) level is also proposed; however, its association with LDL-C is controversial [4]. Some studies found no relationship between TSH and LDL-C in subclinical hypothyroid patients [5, 6]. In contrast, others showed a noticeable rise in LDL-C due to high or even upper-normal levels of TSH [7–10]. Hypothesized mechanisms rationalizing the impact of TSH on the serum level of LDL-C include the high-level expression of 3-hydroxy-3-methylglutaryl-CoA reductase (*HMGCR*) and *PCSK9* genes, as well as repression of bile acid synthesis [11, 12].

TSH induces *HMGCR* transcription through the cyclic adenosine monophosphate/protein kinase A/cyclic adenosine monophosphate-response element binding protein (cAMP/PKA/CREB) pathway, resulting in high-level cholesterol synthesis. High PCSK9 concentration ends up in the diminution of cell surface LDLR and LDL-C uptake capacity [13]. It is noteworthy that some studies remark on a heightened risk of CVD in patients with higher levels of TSH [14, 15]. This point might be even more prominent among a subpopulation of euthyroid patients with FH whose serum TSH is at the upper limit of the normal range. Until now, the association between TSH and LDL-C serum levels in euthyroid patients with FH has not been particularly assessed.

This study is aimed at evaluating the association between TSH levels and LDL-C in euthyroid patients with FH. Moreover, the outcomes may pave the way for other studies to design more efficient therapies.

## 2. Materials and Methods

In this study, the patients were selected from the Isfahan Registry of Familial Hypercholesterolemia (IRFH). This registry was programmed in 2017, aiming to diagnose patients with FH in Isfahan. The complete methodological approach and patient selection have been explained elsewhere [16]. This project was approved by the Research Ethics Committees of the School of Medicine, Isfahan University of Medical Sciences (Approval ID: IR.MUI.MED.REC.1399.784). In brief, the reference laboratories in Isfahan were investigated for patients over two years old with LDL-C levels above 190 mg/dl or above 150 mg/dl but under pharmacological treatment throughout 2017-2021. In addition, patients who were referred to cardiovascular centers due to premature cardiovascular disease (men less than 55 years and women less than 60 years old) were contacted by phone and asked to come to the clinic for further evaluation. Patients with the mentioned characteristics were registered in the IRFH, and the Dutch Lipid Clinic Network Score (DLCNS) was applied to diagnose FH [1].

The criteria consist of family histories, LDL-C levels, and clinical symptoms of FH, such as tendon xanthomas and corneal arcus. Patients were classified into no FH, possible FH, probable FH, and definite FH groups based on their scores. Patients with any cause of secondary hyperlipidemia, such as hypothyroidism, liver and kidney disease, and a drug history of lipid-altering agents, were excluded from the study. Nonetheless, patients on pharmacotherapy for high lipid levels were not excluded. Also, patients with triglyceride (TG) levels above 400 mg/dl were excluded. All the qualified patients participated in the study with complete satisfaction and signed the consent form.

Venous blood samples were collected after the patients had fasted for at least 8 hours overnight. High-density lipoprotein (HDL-C), total cholesterol (TC), and TG levels were measured in serum samples using enzymatic assays (Boehringer, Mannheim, Germany). Serum LDL-C was assessed by the homogenous enzymatic photometric method since it is the most precise way of measuring LDL-C compared to formulas [17]. Serum TSH levels were measured by the DiaZist (Tehran, Iran) enzyme-linked immunosorbent assay (ELISA) kits [18]. TSH's normal reference range was considered 0.45-4.5 mIU/l [19].

In this study, the variables do not follow the normal distribution; therefore, to investigate the association between variables, we utilized nonparametric methods such as the Kruskal-Wallis *H* test and Mann–Whitney *U* Test.

Numeric variables are reported as mean ± standard deviation (SD), and qualitative variables as percentages. Statistical analysis was done using SPSS 24. A *P* value < 0.05 is considered significant.

## 3. Results

The findings from the four-year FH registry are reported elsewhere [20]. A total of 191 patients were included in this study, 25 (13.1%) of whom had definite or probable, and 103 (51.9%) had a possible diagnosis of FH based on DLCN criteria. The demographic data of the participants are shown in Table 1 (see Table 1).

The mean TSH and LDL-C levels among participants were 2.10 ± 1.22 mU/l and 142.17 ± 62.56 mg/dl, respectively. Other lipid profile data of the patients divided by the state of FH diagnosis is demonstrated in Table 2 (see Table 2). As depicted below, the LDL-C and TC levels are significantly higher in the definite/probable FH and possible FH group than in no FH patients (*P* value < 0.0001). The Kruskal-Wallis test was performed on the data to compare the mean TSH levels among the three groups, which showed no significant difference among them (*P* value = 0.87).

In Table 3, possible, probable, and definite FH groups were merged (FH group, *n* = 128), and their LDL-C and TSH levels were compared to no FH patients. The mean LDL-C level was significantly higher in the FH group than in the no FH group (*P* value < 0.0001). However, TSH differences remained insignificant after the combination (*P* value = 0.69) (see Table 3).

Pearson's correlation coefficient was utilized to determine the correlation between TSH and lipid values. No positive or negative correlation was found between serum TSH and TC, LDL-C, TG, and HDL-C. Therefore, according to our data, a change in TSH value does not significantly influence lipid profile (see Table 4).

## 4. Discussion

In the present study, the TSH levels of 191 registered patients in Iran were measured and categorized based on the patient's probability of having FH according to DLCNS criteria. Of 191 patients, 25 had a definite, 103 had a probable or possible FH diagnosis, and 63 were not diagnosed with FH. According to our findings, TSH levels did not differ significantly among the three groups, suggesting that patients with FH have similar TSH levels to the general population. Even after merging the probable FH group into definite FH patients, the absence of a correlation between FH status and TSH levels persists. Our results are consistent with those of a study conducted on 19 patients with FH that indicated no significant difference in TSH levels of FH patients with the control group [21]. There are no further studies on the subject.

We found no correlation between serum TSH levels and TC, LDL-C, HDL-C, and TG in the current study. The mean TSH levels of our 191 samples were 2.10 ± 1.22 mU/l. In contrast to our results, Luxia et al. demonstrated an increase in TC, TG, and LDL-C and a reduction in HDL-C with rising levels of TSH within the normal range [9]. The HUNT study also revealed a positive linear correlation between TSH in the reference range and TC, LDL-C, and TG concentrations and a negative linear correlation with HDL-C [22]. However, a study of 3020 euthyroid participants exhibited a significant linear increase in HDL-C with greater TSH levels. No significant change in LDL-C, TG, and TC was seen, which agreed with our results [23]. To consider TSH levels above normal, in a study conducted by Vierhapper et al., the mean TC, LDL-C, and TG levels of a population of more than 1000 patients with subclinical hypothyroidism were similar to those with TSH within the normal range [6]. On the other hand, a meta-analysis revealed higher TC, LDL-C, and TG levels in subclinical hypothyroid subjects, suggesting that inconsistencies in former studies are affected by confounding factors [24]. In summary, the correlation between lipid profile and serum TSH within the normal range seems controversial among existing literature, and more study on the subject may be required.

The limitations of this study include a relatively small sample size and a lack of genetic testing to diagnose FH. However, this is one of the first studies on thyroid abnormalities in euthyroid patients with FH. More research in this field will pave the way to developing new medications to treat these patients, especially thyroid hormone receptor agonists.

## 5. Conclusion

We found no correlation between serum TSH levels and lipid profiles in euthyroid patients with FH.

## Figures and Tables

**Table 1:** Demographic data of the participants.

	Total (*N* = 191)
Male sex, % (*N*)	56 (107)
Age, mean ± SD, years, range	46.66 ± 18.53 (2-85)
BMI, mean ± SD (kg/m^2^)	27.05 ± 5.16
Weight, mean ± SD (kg)	71.85 ± 18.18
Height, mean ± SD (cm)	162.20 ± 17.28
BMI, % (*N*)	
Normal (<25 kg/m^2^)	29.3 (56)
Overweight (25-29.9 kg/m^2^)	42.9 (82)
Obese (≥30 kg/m^2^)	24.6 (47)
Waist, mean ± SD (cm)	96.20 ± 15.25
Hip, mean ± SD (cm)	104 ± 12.37
Smoking status, % (*N*)	
Current	9.9 (19)
Past	3.7 (7)
Never	86.4 (165)
Smoking, mean ± SD, pack/year	11.32 ± 11.09
CAD (MI, STEMI, NSTEMI, Angina), % (*N*)	14.1 (27)
Valvular heart disease, % (*N*)	3.7 (7)
Hypertension, % (*N*)	32.3 (61)
Arcus cornealis, % (*N*)	3.7 (7)
Xanthoma, % (*N*)	3.1 (6)
Xanthelasma, % (*N*)	1.1 (2)
Family history of premature CVD, % (*N*)	37 (71)
Antihypertensive drug use, % (*N*)	55.5 (106)
Lipid-lowering drug use, % (*N*)	80.1 (153)
Diastolic blood pressure, mean ± SD (mmHg)	84.16 ± 11.03
Systolic blood pressure, mean ± SD (mmHg)	131.86 ± 19.77
HbA1c, mean ± SD, %	6.18 ± 2.24

BMI: body mass index; kg: kilograms; cm: centimeters; CVD: cardiovascular disease; CAD: coronary artery disease; MI: myocardial infarction; STEMI: ST-elevation myocardial infarction; NSTEMI: non-ST-elevation myocardial infarction; mmHg: millimeters of mercury; HbA1C: hemoglobin A1C.

**Table 2:** TSH and lipid profile of the participants.

Variables	Total (*n* = 191)	No FH (*n* = 63)	Possible FH (*n* = 103)	Probable and definite FH (*n* = 25)	*P* value^∗^
TSH (mU/l)	2.10 ± 1.22 (0.3-5.5)	2.2 ± 1.37	2.03 ± 1.13	2.12 ± 1.19	0.87
LDL-C (mg/dl)	142.17 ± 62.56	108.25 ± 32.56	144.82 ± 49.22	214.08 ± 96.78	<0.0001^∗∗∗^
HDL-C (mg/dl)	49.46 ± 10.80	49.53 ± 11.46	48.42 ± 10.05	53.60 ± 11.54	0.098
TC (mg/dl)	220.91 ± 74.53	181.63 ± 40.70	221.63 ± 57.78	312.24 ± 111.26	<0.0001^∗∗∗^
TG (mg/dl)	173.61 ± 142.39	166.63 ± 190.95	180.13 ± 118.27	163.76 ± 90.74	0.078

**Table 3:** Mean LDL-C and TSH levels and comparison between FH and no FH groups.

	Total (*n* = 191)	FH		*P* ^∗^
		No FH (*n* = 63)	FH (*n* = 128)	
LDL-C (mg/dl)	142.17 ± 62.56	108.25 ± 32.56	158.34 ± 66.90	<0.0001^∗∗∗^
TSH (mU/l)	2.10 ± 1.22	2.2 ± 1.37	2.05 ± 1.14	0.69

**Table 4:** Correlation between TSH and lipid profile.

		LDL-C	HDL-C	TC	TG
TSH	Pearson's correlation	0.093	0.088	0.057	0.013
	*P* value	0.203	0.225	0.438	0.863

## Data Availability

The data used to support the findings of this study are available from the corresponding authors upon request.

## References

[1] Najam O., Ray K. K. (2015). Familial hypercholesterolemia: a review of the natural history, diagnosis, and management. *Cardiology and therapy*.

[2] Bouhairie V. E., Goldberg A. C. (2015). Familial hypercholesterolemia. *Cardiology Clinics*.

[3] Singh S., Bittner V. (2015). Familial hypercholesterolemia--epidemiology, diagnosis, and screening. *Current Atherosclerosis Reports*.

[4] Rizos C. V., Elisaf M. S., Liberopoulos E. N. (2011). Effects of thyroid dysfunction on lipid profile. *Open Cardiovascular Medicine Journal*.

[5] Hueston W. J., Pearson W. S. (2004). Subclinical hypothyroidism and the risk of hypercholesterolemia. *Annals of Family Medicine*.

[6] Vierhapper H., Nardi A., Grösser P., Raber W., Gessl A. (2000). Low-density lipoprotein cholesterol in subclinical hypothyroidism. *Thyroid*.

[7] Althaus B. U., Staub J. J., Lèche A. R., Oberhänsli A., Stähelin H. B. (1988). LDL/HDL-changes in subclinical hypothyroidism: possible risk factors for coronary heart disease. *Clinical Endocrinology*.

[8] Witte T., Ittermann T., Thamm M., Riblet N. B., Völzke H. (2015). Association between serum thyroid-stimulating hormone levels and serum lipids in children and adolescents: a population-based study of german youth. *The Journal of Clinical Endocrinology and Metabolism*.

[9] Luxia L., Jingfang L., Songbo F. (2021). Correlation between serum TSH levels within normal range and serum lipid profile. *Hormone and Metabolic Research*.

[10] Efstathiadou Z., Bitsis S., Milionis H. J. (2001). Lipid profile in subclinical hypothyroidism: is L-thyroxine substitution beneficial?. *European Journal of Endocrinology*.

[11] Gong Y., Ma Y., Ye Z. (2017). Thyroid stimulating hormone exhibits the impact on LDLR/LDL-c via up- regulating hepatic PCSK9 expression. *Metabolism*.

[12] Song Y., Xu C., Shao S. (2015). Thyroid-stimulating hormone regulates hepatic bile acid homeostasis via SREBP-2/HNF-4*α*/CYP7A1 axis. *Journal of Hepatology*.

[13] Tian L., Song Y., Xing M. (2010). A novel role for thyroid-stimulating hormone: up-regulation of hepatic 3-hydroxy-3-methyl-glutaryl-coenzyme a reductase expression through the cyclic adenosine monophosphate/protein kinase A/cyclic adenosine monophosphate–responsive element binding protein pathway. *Hepatology*.

[14] Inoue K., Ritz B., Brent G. A., Ebrahimi R., Rhee C. M., Leung A. M. (2020). Association of subclinical hypothyroidism and cardiovascular disease with mortality. *JAMA Network Open*.

[15] Duntas L. H., Chiovato L. (2014). Cardiovascular risk in patients with subclinical hypothyroidism. *European endocrinology*.

[16] Vaseghi G., Arabi S., Haghjooy-Javanmard S. (2019). CASCADE screening and registry of familial hypercholesterolemia in Iran: rationale and design. *ARYA atherosclerosis*.

[17] Vaseghi G., Rezvanian P., Taheri M. (2022). Comparison of equations for the calculation of LDL-cholesterol in familial hypercholesterolemia: data from Iranian registry. *Türk Kardiyoloji Derneği Arşivi*.

[18] Vaseghi G., Heshmat-Ghahdarijani K., Taheri M. (2022). Hematological inflammatory markers in patients with clinically confirmed familial hypercholesterolemia. *BioMed Research International*.

[19] Rugge J. B., Bougatsos C., Chou R. (2015). Screening and treatment of thyroid dysfunction: an evidence review for the U.S. preventive services task force. *Annals of Internal Medicine*.

[20] Vaseghi G., Taheri M., Heshmat-Ghahdarijani K. (2021). Familial hypercholesterolemia (FH) in Iran: findings from the four-year FH registry. *Journal of Lipids*.

[21] Vessby B., Wide L. (1975). Serum levels of thyroid-stimulating hormone in hyperlipoproteinemia. *Clinica Chimica Acta*.

[22] Asvold B. O., Vatten L. J., Nilsen T. I., Bjøro T. (2007). The association between TSH within the reference range and serum lipid concentrations in a population-based study. The HUNT study. *European journal of endocrinology*.

[23] Ahi S., Amouzegar A., Gharibzadeh S. (2017). The association between normal range TSH and lipid profile. *Hormone and Metabolic Research*.

[24] Treister-Goltzman Y., Yarza S., Peleg R. (2021). Lipid profile in mild subclinical hypothyroidism: systematic review and meta-analysis. *Minerva endocrinology*.

